# Suicide Prevention as a Pillar of Sustainable Mental Health: A Focused Comparative Narrative Review of the Republic of Cyprus and Selected European Countries in the Post-COVID-19 Era

**DOI:** 10.3390/healthcare14111528

**Published:** 2026-06-01

**Authors:** Maria Karanikola, Nicos Middleton

**Affiliations:** Department of Nursing, School of Health Sciences, Cyprus University of Technology, 3036 Limassol, Cyprus; nicos.middleton@cut.ac.cy

**Keywords:** suicide prevention, sustainable development, mental health policy, COVID-19, European Union, public health, gender disparities, health systems, national strategies

## Abstract

**Highlights:**

**What are the main findings?**

- Suicide prevention strategies across selected European countries demonstrate heterogeneous levels of development and implementation, with substantial variation in national mental health policies, surveillance frameworks, and monitoring mechanisms.
- The review highlights the importance of robust epidemiological surveillance systems and improved data comparability as essential foundations for evidence-informed suicide prevention policies.
- Suicide patterns appear to be associated with multifactorial determinants, including demographic characteristic—mainly age and gender, socioeconomic conditions, and differing national policy responses across countries.

**What are the implications of the main findings?**

- Strengthening suicide prevention across European settings requires coordinated, long-term, cross-sectoral, and gender-sensitive national strategies.
- Investment in standardized surveillance systems and high-quality epidemiological data infrastructure is essential for evidence-based policy development and evaluation.
- Sustainable progress in suicide prevention requires alignment with broader public health and long-term mental health policy frameworks.

**Abstract:**

**Background/Objectives:** Mental health and suicide prevention are increasingly recognized as critical components of sustainable development in the European Union (EU), particularly in light of the broader mental health challenges highlighted during the COVID-19 pandemic. This review aimed to explore suicide prevention policies and mental health strategies across selected European countries through a focused comparative analysis centered on the Republic of Cyprus. **Methods:** A narrative review design was applied. A purposive literature search focused on national strategies, epidemiological trends, policy papers, and peer-reviewed articles published from 2000 to January 2026 was followed. Databases searched included PubMed, Scopus, PsychInfo, Embase and Google Scholar, supplemented by grey literature from the World Health Organization (WHO), European Commission, and national health authorities. The review focused on selected European countries, i.e., the United Kingdom, Sweden, Finland, Greece, and the Republic of Cyprus, chosen to illustrate variation in suicide prevention policies, health system structures, and implementation frameworks. Evidence was critically appraised and synthesized thematically to identify commonalities and contrasts in policy, implementation and emerging challenges. **Results:** The review identified substantial variation in national suicide prevention strategies, monitoring systems, and policy implementation across the selected countries. Persistent gender- and age-related disparities in suicide patterns were observed, alongside the influence of socio-economic determinants, and the broader mental health effects associated with the COVID-19 pandemic. The findings also underscored the need for robust, gender-sensitive, and data-driven national strategies that are contextually grounded and equitably resourced. **Conclusions:** This review concludes with recommendations for enhancing mental health sustainability across Europe, emphasizing cross-sectoral coordination, improved surveillance systems, and future research priorities.

## 1. Introduction

Mental health is a cornerstone of sustainable development, while suicidality continues to be a major public health concern across the European Union (EU) [1,2]. The COVID-19 pandemic exposed and exacerbated existing vulnerabilities in mental health care systems [3], while intensified concerns about population mental health [4], and renewed policy attention to suicide prevention as a central goal of public health planning [5]. Indeed, the relevance of suicide prevention to sustainable development is underscored by its inclusion in Goal 3 of the United Nations (UN) Sustainable Development Goals (SDGs), which addresses “Good Health and Well-being”. The target of Goal 3 primarily tracks suicide mortality rates, since these rates represent an important population-level indicator of overall mental health, particularly in the absence of other routinely collected epidemiological data with nationwide geographical coverage [6].

National suicide prevention programs began to emerge in the 1990s as a policy response to the growing recognition of suicidality as a complex and preventable public health issue [7]. To date, approximately 29 countries have developed national strategies, mostly aiming at identifying vulnerable groups, improving care for those with self-injurious behaviour, and enhancing research and surveillance systems [8]. Such programs also focus on raising awareness and reducing stigma associated with suicidality and mental disorders [9]. International organizations, such as the World Health Organization (WHO) and the International Association for Suicide Prevention (IASP) have developed guidelines, emphasizing the need for: (a) addressing both suicide and self-injury (suicidal and non-suicidal), (b) strengthening support and rehabilitation services for at-risk individuals, (c) mobilizing national governments to scaling up financial and technical support, (d) establishing measurable objectives, accompanied by systematic and methodologically robust research and monitoring frameworks [9].

Building on these approaches, a range of recommendations and policy initiatives aiming to prevent suicidality as part of broader mental health and wellbeing promotion strategies have continued to evolve in the post-pandemic era [10]. Nevertheless, although not uniformly, there is data linking the COVID-19 crisis with intensified psychological distress, anxiety and depressive symptoms, social isolation, and economic insecurity [5,11]. Additionally, the pandemic has underscored the importance of resilient mental health systems and coordinated prevention planning [3]. Importantly, although suicide mortality remains complex and heterogeneous across countries and time periods, it is encompassed as a central pillar in national mental health strategic planning [5,11].

Similarly suicide prevention has gained increasing prominence within European health policy frameworks; yet comparative literature remains fragmented with respect to national strategies, surveillance infrastructures, and policy responses to emerging challenges such as the COVID-19 pandemic. Existing reviews frequently provide broad overviews of European developments but often offer limited attention to smaller or under-represented policy contexts. Accordingly, a focused, comprehensive synthesis examining selected European countries, while situating the Republic of Cyprus (RC) within broader European developments, may help illuminate both shared policies and context-specific implementation gaps relevant to sustainable mental health planning. Examining diverse mental health practices and suicide prevention policies across selected European countries in the period surrounding and following the COVID-19 pandemic is expected to provide an opportunity to assess whether policy responses supported progress towards improved mental health and wellbeing within the broader context of sustainable development.

The present review aimed to conduct a focused comparative narrative analysis of suicide prevention strategies across selected European countries, using the RC as the primary analytical case situated within a broader European policy context. Particular attention was given to developments occurring during and after the pandemic, differences in surveillance systems, demographic vulnerabilities, and emerging prevention approaches across selected national settings.

## 2. Materials and Methods

### 2.1. Conceptual Orientation and Design of the Review

The study adopted a focused comparative narrative review methodology aiming to synthesize and interpret existing literature on suicide epidemiology and prevention strategies across the RC and selected European countries. Narrative reviews are particularly suited for examining complex public health issues that involve heterogeneous sources of evidence, including epidemiological studies, policy analyses, and program evaluations [12]. Unlike systematic or scoping reviews that apply rigid protocols and quantitative aggregation of findings, narrative reviews allow for a broader interpretive integration of diverse types of evidence and conceptual perspectives [13]. Thus, narrative reviews are widely used in public health research when the aim is to contextualize evidence and explore policy and system-level developments across multiple settings [12,13,14].

This approach was considered appropriate because suicide prevention policies in Europe involve multiple dimensions, including demographic trends, behavioral risk factors, health system responses, and policy implementation mechanisms [15]. The narrative review design therefore enabled the integration of empirical findings with policy documentation and surveillance reports in order to provide a comprehensive understanding of how suicide prevention strategies have evolved across European countries, particularly in relation to healthcare and societal challenges associated with the COVID-19 pandemic.

More specifically, this narrative review adopted a focused comparative approach examining a subset of European countries rather than attempting exhaustive coverage of all EU member states. The RC was used as the primary analytical case, while incorporating selected comparisons with other European countries to contextualize suicide prevention policies, epidemiological patterns, and surveillance practices across differing health system and institutional settings. The emphasis on the RC was intentional and reflected its evolving suicide prevention policy landscape, limited surveillance infrastructure, and relative underrepresentation within comparative European suicide prevention literature. Consequently, the review was not intended to provide a comprehensive assessment of all European countries, but rather an interpretive comparative synthesis of selected European experiences relevant to the study objectives.

Countries were selected purposively based on: (1) the availability of publicly documented national suicide prevention strategies or mental health policy frameworks; (2) the accessibility of epidemiological and policy evaluation data in English-language or internationally accessible sources; (3) geographical diversity across Northern, Western, and Southern Europe; and (4) the relevance of selected countries as illustrative examples of differing health system structures and suicide prevention approaches.

Based on these criteria, the review examined the United Kingdom, Sweden, Finland, Greece, and the RC as primary case examples, while also referencing broader European policy developments where relevant. This approach allowed for in-depth qualitative comparison of policy frameworks, implementation mechanisms, and monitoring practices within different institutional and socio-economic contexts.

### 2.2. Aim and Research Questions

The aim of the present narrative review was twofold: (i) to describe and compare national suicide prevention strategies within selected European countries, and (ii) to examine demographic, temporal, behavioral, and contextual factors associated with suicidality, before and after the COVID-19 pandemic. Within this framework, the review sought to synthesize epidemiological evidence together with policy developments to identify patterns, risk determinants, and prevention priorities across the region.

Accordingly, the review focused on the following objectives:

(A) Description of gender- and age-specific patterns of suicide, also giving emphasis on adolescents and young adults, who have been identified as a priority group in several prevention frameworks.
(B) Examination of gender differences in suicide methods and their implications for means-restriction and other evidence-based prevention strategies.
(C) Identification of seasonal and temporal variations in suicide rates and fluctuations across demographic groups.
(D) Exploration of the relationship between non-suicidal self-injury, substance use, and suicide risk.
(E) Assessment of the contribution of surveillance systems, data quality, and media practices to the design and effectiveness of suicide prevention strategies.

These objectives collectively guided the selection, analysis, and synthesis of the relevant evidence.

### 2.3. Literature Search Strategy

Τhe search strategy was designed to identify key and representative sources relevant to the objectives of the review rather than to achieve exhaustive coverage of all publications on suicide and suicide prevention in Europe. This approach is consistent with narrative review methodology, which prioritizes conceptual integration and policy interpretation over comprehensive evidence mapping [12,13,14]. Specifically, a structured but purposive literature search was employed to identify relevant scientific publications, epidemiological data sources and policy reports published between 2000 to January 2026. This timeframe was chosen because the early 2000s marked a period during which several European countries began developing structured national suicide prevention strategies and strengthening mental health policy frameworks. Additionally, the selected period captures recent developments related to the COVID-19 pandemic and its potential psychosocial consequences.

Databases searched included PubMed, Scopus, PsychInfo, Embase and Google Scholar. Search terms were developed based on the research objectives and included combinations of keywords related to suicide epidemiology and prevention policies, and included: suicide, suicidal behaviour, suicide prevention, self-harm, non-suicidal self-injury, substance use, suicide methods, gender differences, youth suicide, seasonality, European Union/Europe and national suicide prevention strategies. Boolean operators (AND, OR) were applied to refine the search and identify relevant combinations of terms. This search was last updated in January 2026.

In addition to peer-reviewed literature, grey literature sources were consulted in order to capture policy documents and official reports that are often not indexed in academic databases. These included publications from international organizations, European institutions, and national public health authorities. Particular attention was given to documents addressing suicide monitoring systems, policy frameworks, and post-pandemic mental health responses. The search strategy also incorporated reference list screening and citation tracking of key publications in order to identify additional relevant sources.

### 2.4. Study Selection and Eligibility Criteria

Relevant sources identified through the search process were assessed for relevance based on predefined inclusion and exclusion criteria.

Inclusion criteria comprised: (a) peer-reviewed empirical studies or systematic analyses addressing suicide or suicidal behaviour in European populations, (b) publications examining suicide prevention policies, national strategies, surveillance systems, or public health interventions within selected European countries, particularly where findings contributed to comparative understanding relevant to the RC and broader European suicide prevention discussions, (c) studies focusing on demographic patterns, suicide methods, temporal trends, or associated behavioural risk factors, (d) reports and policy documents from reputable governmental or international organizations, and (e) publications written in English and published between 2000 and January 2026.

Sources focused exclusively on non-European contexts without relevance to European policy discussions, unless they provided relevant comparative insights for European policy development, as well as publications lacking empirical, epidemiological, or policy relevance were excluded, as were duplicated records or sources with insufficient methodological information.

Following initial identification of relevant literature, titles and abstracts were assessed to confirm relevance to the review objectives. Full texts of potentially relevant publications were subsequently assessed to confirm their suitability in the narrative synthesis.

### 2.5. Data Extraction, Organization and Synthesis Approach

Relevant information from the selected literature was extracted and organized to facilitate comparative analysis. Specifically, data were organized in thematic matrices to support systematic comparisons across countries and to identify similarities, differences, and policy trends [12]. This process facilitated the integration of epidemiological findings with policy-level evidence across the reviewed sources.

Data synthesis followed a deductive thematic narrative synthesis approach [13]. The analytical framework was defined a priori and directly derived from the study objectives, and data were subsequently organized and interpreted within these pre-specified thematic domains rather than allowing themes to emerge inductively from the data. Accordingly, the analysis focused on the following predefined thematic domains: (a) demographic and gender differences in suicide patterns, (b) suicide methods and implications for means restriction policies, (c) seasonal and temporal variations in suicide rates, (d) behavioral risk factors such as non-suicidal self-injury and substance use, (e) structural and policy-related determinants, including surveillance systems and media reporting practices. No additional themes were generated during analysis beyond the predefined analytical framework. This thematic narrative synthesis approach is commonly applied in narrative reviews addressing complex public health or policy issues where evidence sources are heterogeneous and not readily amenable to quantitative synthesis [14].

Through this structured process, the review comparatively examined epidemiological trends, suicide prevention policies, and surveillance practices across selected European countries, using the RC as principal contextual reference point within the predefined analytical framework. Particular attention was given to changes observed before and after the COVID-19 pandemic, as this period has been associated with substantial social, economic, and mental health challenges. Moreover, the analysis focused on identifying similarities and differences in national policy frameworks, surveillance systems, and implementation mechanisms, and the integration of gender-sensitive and population-specific interventions. This comparative perspective allowed the review to explore how differing institutional contexts, health system structures, and policy infrastructures may shape suicide prevention approaches across selected European settings. Although the synthesis was guided by predefined domains, policy documents were interpreted within their institutional and public health contexts, allowing the synthesis to capture not only reported interventions but also broader strategic priorities guiding suicide prevention efforts and ensuring contextual sensitivity.

Consistent with narrative review methodology [12,13,14], this manuscript adopted an integrated results–discussion structure. Within this format, findings are presented together with contextual interpretation in thematic subsections, enabling a continuous synthesis of evidence and facilitating the contextualization of epidemiological and policy-related insights across the reviewed literature.

### 2.6. Ensuring Transparency and Methodological Rigor

Although narrative reviews do not follow the strict protocols of systematic reviews, several measures were implemented to enhance transparency and methodological rigor. These included the explicit definition of research objectives, the use of multiple academic databases, the application of predefined eligibility criteria, and the inclusion of both peer-reviewed and grey literature sources.

Furthermore, the synthesis also involved critical appraisal of the included evidence, taking into account methodological limitations of individual studies, differences in national data collection systems, and variations in reporting practices across countries. By combining epidemiological data with policy documentation and surveillance reports, the review aimed to provide a contextualized comparative overview of selected suicide prevention efforts within European settings relevant to the review objectives.

While structured search procedures and predefined eligibility criteria were used to improve transparency and reduce potential selection bias, since the review was not designed as a systematic or scoping review and therefore, it did not follow formal reporting frameworks such as PRISMA. Rather, consistent with established narrative review methodology [12,13], the objective was to provide an interpretive and context-sensitive comparative synthesis of heterogeneous epidemiological, policy, and surveillance evidence relevant to suicide prevention in selected European settings, with particular emphasis on the RC as a focal comparative case. Subsequently, a table presenting the principal evidence sources included in the narrative review and their contribution to the comparative synthesis was developed as Table S1 (Supplementary Material). It is clarified that the data presented in this table are selective and illustrative, consistent with the focused comparative narrative design of the review. This table summarizes the principal categories of evidence informing the thematic synthesis rather than providing an exhaustive inventory of all included sources.

## 3. Results and Discussion

### 3.1. National Strategies Towards Suicide Prevention

#### 3.1.1. Empirical Evidence from the Selected Countries

In the United Kingdom (UK), the policy paper “Suicide Prevention in England: 5-year Cross-sector Strategy” was published in September 2023 [16]. The goal of this strategy was the reduction in suicide deaths through coordinated cross-sectoral actions over the 5-year period, beginning in 2023. The areas of focus of this strategy included: (a) improving data and evidence to inform effective interventions, (b) providing personalized and targeted support to priority groups, (c) addressing risk factors, (d) online safety, (e) technology and media, (f) providing appropriate and effective crisis support, (g) tackling methods and means of suicide, (h) providing timely and effective support to suicide-bereaved survivors.

Similarly, the Swedish new national strategy for mental health and suicide prevention contained nine strategic areas of action with relevant goals and indicators for follow-up [17]. Equally, the Finnish Institute for Health and Welfare published the national “Suicide Prevention Program for 2020–2030”, which contains 36 objectives and related actions, aiming to prevent suicide deaths. The implementation and effectiveness of the actions encompassed in each objective are monitored according to relevant indicators [18]. The main topics are (a) awareness raising, (b) impacting the means of suicide, (c) early intervention, (d) supporting risk groups, (e) developing care options, (f) increasing media competence, (g) strengthening knowledge basis and research, (h) monitoring of the suicide prevention program and proposals for indicators.

An overview of national efforts towards suicide prevention in the Nordic countries (Denmark, the Faroe Islands, Finland, Greenland, Iceland, Norway, Sweden and Åland) has been recently published and illustrates the strong institutional integration of surveillance systems and prevention programs within broader public health frameworks [19].

In the RC, a national strategy for mental health is currently under development, with the support of the newly established WHO country office. The process follows a participatory approach involving key stakeholders, including people with living experience (PLEs) and caregivers, an approach that remains relatively uncommon in the national context of the RC. In parallel, recent epidemiological research has also provided important evidence to inform suicide prevention policy in the RC. Specifically, Chatzittofis et al. [20] examined trends in suicide mortality rates prior to the COVID-19 pandemic and provided the first systematic analysis of suicide patterns in the country. Their findings highlighted concerning trends and identified important gaps in suicide prevention infrastructure and policy development in the country. Moreover, the study drew attention to targeted prevention measures for high-risk groups and availability of resources within the specific legal and cultural context of the RC.

#### 3.1.2. Interpretive Synthesis

It seems that national suicide prevention strategies have increasingly been developed across Europe in response to recommendations from the International Association for Suicide Prevention and the World Health Organization [15]. Selected European countries have adopted national frameworks or action plans for suicide prevention, while in some countries, including Greece and the RC, such strategies are under development. The comparative findings illustrate considerable diversity in the development and implementation of suicide prevention strategies across selected European countries, reflecting differences in policy maturity, surveillance capacity, and stakeholder engagement. While some countries have established comprehensive policy frameworks with defined monitoring indicators, others remain at earlier stages of strategy development and surveillance integration. This heterogeneity reflects broader differences in institutional capacity, mental health infrastructure, and public health across European settings.

### 3.2. Demographic, Temporal, Behavioral, and Contextual Factors Associated with Suicidality in Europe Before and After the COVID-19 Pandemic

#### 3.2.1. Suicide by Gender and Age

##### Empirical Evidence from the Selected Countries

Evidence across Europe consistently shows substantial gender differences in suicide mortality, with suicide rates consistently higher among men than women across the selected European countries.

Suicide rates in the RC were found to be approximately four times higher in men than in women, with violent methods accounting for around 80% of suicides in both genders [20]. During the pre-pandemic study period, male suicide rate doubled between 2004 and 2012 before declining after 2013 to 2020. However, this decline was not uniform across all age groups, with suicide rates continuing to rise among men aged 45–64 [20].

Regarding female suicide patterns, Chatzittofis et al. [20] identified a declining trend in female suicide rates in the RC during the study period (2004–2020), contrasting with earlier recorded upward trend between 1988 and 1999. While the reasons for this decline are unclear, this reflects the well-established gender differentials in suicide trends. The highest suicide rates among females were recorded in the 45–64 age group, followed by the 25–44 age group. However, the relatively smaller number of female suicides in a country the size of the RC limits the ability to draw inferences about the observed differences and trends. Unfortunately, the study period did not include the pandemic years; thus, it is not clear whether the previously declining trend in females continued or reversed during the pandemic or in the post-pandemic years. While it would be important to monitor suicide trends in the subsequent period, it should be noted that delays in recording systems may be longer in the RC than elsewhere. Even though this is a universal phenomenon to a larger or lesser degree, it is highly dependent on death certification, coroner and registration practices in each country.

In England and Wales, the highest suicide rates registered in 2023 occurred among men aged 45 to 64 years, representing the highest rates recorded across all age groups since 2010 [21]. These findings highlight age- and gender-specific variation in suicide mortality patterns.

Similar age-specific patterns have been observed internationally. Research examining the effects of the 2008 global financial crisis found an increase in suicide rates, especially in middle-aged men in several countries [22]. Economic stressors, such as job loss and unemployment, debt, and financial insecurity have been identified as contributing factors, particularly among men in mid-life who often carry substantial family and economic responsibilities [22,23]. In Greece, which was also severely affected by the 2008 financial crisis, male suicide rates increased by 22% between 2007 and 2011, with the highest increases occurring in men aged 45–64 [24].

However, these patterns were not observed uniformly across European countries. In Austria, comparable increases were not identified [22], where it is suggested that stronger social and mental health protection policies may potentially have moderated the impact of economic instability on suicide mortality. Similarly, evidence from Sweden and Finland on suicide mortality rates in times of financial crisis may also suggest that active labor market programs combined with interventions that promote access to mental health services may mitigate the impact of economic downturns on suicide risks [22].

Economic and employment-related stressors also emerged during the COVID-19 pandemic and remain in the post-COVID-19 period [25,26]. Financial insecurity and employment disruptions were widely reported in the UK, especially among vulnerable populations [25]. Studies linked financial difficulties during the pandemic with symptoms of mental health problems [25]. Long COVID was additionally associated with employment instability and financial disruption, suggesting that employment protection and financial support may play an important role in mitigating further physical and mental health deterioration in people with long COVID [26].

Financial support measures and employment protection programs were introduced by the selected European countries during the pandemic. The “Living with COVID” and the “Coronavirus Statutory Sick Pay Rebate Scheme” programs were implemented by the UK government to address these issues. Other programs introduced during the pandemic targeting recovery included the “Kickstart scheme”, the “Recovery Loan Scheme” and the “Job Entry Targeted Support program”. Additionally, a unique package to support the lowest-paid and unemployed individuals was further introduced, including the “Coronavirus Job Retention Scheme” and the “Self-Employment Income Support Scheme” [27]. Similar initiatives to provide debt relief and access to financial support for individuals facing severe economic hardships were launched in other European countries with the support of the European Commission, such as Germany and Denmark, where targeted financial and mental health support programs were directed at at-risk groups, considering gender and age [28].

Regarding mental health initiatives implemented during the pandemic to support women, international data showed that these primarily focused on survivors of intimate partner and domestic violence, while any of these initiatives relied on a wide range of technology-based solutions [29].

##### Interpretative Synthesis

In relation to the provision of annual mortality reports, It should be noted that although this is a routine in several countries is not routine practice in other countries, such as in the RC. Data on deaths by suicide in the RC are recorded by the Health Monitoring Unit as part of the Death Registry, but these are neither openly available to the research community nor included in annual Mortality Reports by the Cyprus Ministry of Health at a disaggregated and meaningful level. Data are only available by request and, thus, the responsibility for monitoring suicide trends and patterns falls to the local research community [20]. This organizational arrangement may contribute to delays in data accessibility and may further widen the gap between research activity and public health practice. The National Suicide Strategy for Mental Health currently under development may address some of these limitations and contribute to improved coordination of suicide surveillance, prevention activities and accessibility in the RC.

#### 3.2.2. Suicide Methods by Gender

##### Empirical Evidence from the Selected Countries

Research across Europe indicates substantial variation in suicide methods by gender.

Consistent with international trends, hanging is currently the most common suicide method in males, replacing self-poisoning, self-shooting, and other methods that were more prevalent in previous decades [20,30]. The increasing predominance of hanging as a suicide method in many European countries has been discussed in relation to suicide prevention efforts aimed at reducing access to firearms and pesticides [31,32]. Suicide deaths by poisoning declined in the UK following the introduction of pesticide control legislation in the 1990s [21]. However, hanging remains the most prevalent suicide method in the post-COVID-19 period in the UK as well as other European countries [21].

Chatzittofis et al. [20] reported that self-shooting remained the second most common suicide method in males in the RC. The authors associated this finding with the widespread availability of firearms within the country, as well as militarization of the RC. In contrast, no women suicides involving firearms were reported in the RC during the study period, despite comparable access to these weapons; this may suggest potential socio-cultural or biological and behavioural differences in the choice of violent methods.

Several European countries have implemented means-restriction measures targeting highly lethal suicide methods. Austria and Germany introduced restricted access to firearms, while Switzerland implemented restrictions involving both lethal pesticides and firearms [32]. These interventions were largely developed in response to observed male suicide patterns.

In contrast, fewer interventions have specifically addressed suicide patterns among females across the selected European countries. The UK implemented measures including paracetamol sales restrictions to reduce medication overdose deaths [33]. However, violent suicide methods, including hanging and jumping, were also identified as the most common suicide methods in women in the RC [20], challenging assumptions that women typically use less violent methods, such as drug overdose. Similar patterns have been reported in other European countries, where a growing number of women have been reported to choose violent suicide methods, including hanging and jumping, in recent years [34].

Available data on gender-specific suicide prevention interventions for women remains limited [32]. The majority of related interventions include psychotherapeutic programs on adaptive coping and help-seeking behaviors, mental health screening programs, stigma-reduction campaigns, targeted support programs for identifying and supporting those at higher risk, digital platforms and hotlines offering anonymous support.

##### Interpretive Synthesis

The findings from the selected European countries suggest that suicide methods are shaped by an interaction between access to means, socio-cultural factors, and gender-related behavioral patterns. The evidence also indicates evolving method profiles in both men and women across the selected European settings.

Current means-restriction policies in European countries appear to focus mainly on male suicide patterns, likely reflecting persistently higher male suicide rates. At the same time, emerging evidence regarding the increasing use of violent suicide methods among females suggests changing behavioural patterns that remain insufficiently examined within existing literature.

Overall, the present comparative findings from the selected European countries underscore the importance of considering gender, social, and cultural context in the interpretation of suicide method patterns and in the development of suicide prevention programs. Research examining gender-specific patterns and the effectiveness of relevant interventions, particularly in women, remains limited across the selected European contexts [32].

#### 3.2.3. Seasonal Trends, Gender and Age Groups

##### Empirical Evidence Form Selected Countries

Seasonality and related temporal factors have been incorporated into suicide prevention strategic planning in some countries.

A seasonality pattern in suicide mortality was identified in the RC, with male suicides occurring more frequently in the spring and summer months [20], a pattern also observed in other Northern Hemisphere countries. In Sweden and Norway, mental health programs that specifically target vulnerable populations during high-risk months for suicide, such as spring and summer, have been established [19]. These initiatives often include increased awareness campaigns and increased accessibility to support services during these periods. The national action program for suicide prevention introduced in Sweden in 2008 includes specific strategies for addressing seasonal variations in suicide risks [11]. This includes collaboration across various governmental and non-governmental organizations in the provision of mental health support during the high-risk periods [19].

In France, suicide prevention campaigns addressing seasonal spikes in suicide rates have also been implemented [35]. These efforts focus on increasing public awareness and providing support resources, particularly during the spring and summer months, when suicide rates tend to increase. These measures form part of a broader national effort to align mental health service provisions with seasonal patterns in suicide risk, and ensure that vulnerable groups, and especially those with a previous suicide attempt history, receive the necessary support [35].

Several studies have explored explanations for seasonal variation in suicide mortality, including bioclimatic factors, such as sunlight exposure, or changes in social and outdoor activity during particular months. Research has also examined interventions involving outdoor activity and environmental exposure with broader approaches addressing seasonal mental health variation [36].

##### Interpretive Synthesis

Evidence from the selected countries suggests that environmental and seasonal factors may contribute to temporal fluctuations in suicide risk, with several European countries reporting consistent seasonal variation in suicide mortality patterns. The comparative findings further indicate that epidemiological evidence may inform the development and coordination of targeted prevention strategies and service provision in selected national contexts.

#### 3.2.4. Suicide in Young People

##### Empirical Evidence for Selected Countries

Although suicide rates in individuals under 20 years remain lower than those observed in older age groups [1,2], selected European countries have reported increasing trends in youth suicide over the past decade.

Evidence from the United Kingdom indicates that suicide rates among young people increased in the late 2010s, with a particularly notable increase observed among females under the age of 25 in England and Wales [21]. Similar patterns have been reported in other European settings, where surveillance data suggest growing psychological distress and self-harm behaviours, including suicide mortality, among adolescents and young adults, particularly among young women [37].

Comparative evidence from outside Europe also illustrates the broader international dimension of these concerns. Data from the United States indicate that the proportion of adolescent females reporting suicidal ideation increased substantially during the pandemic period, with approximately 30% reporting that they had seriously considered suicide in 2021 [38]. Although these findings originate from a non-European context, they provide a broader perspective on mental health vulnerabilities observed among young populations during the same period.

To address the rise in youth mental health challenges, England incorporated targeted actions within national mental health policies focused on strengthening early detection and support for adolescents and young adults [21]. These initiatives included expanded funding for school-based mental health programs, designed to facilitate early detection of mental health issues, alongside community-based initiatives that extended support beyond conventional healthcare environments. Digital outreach was also a key component, with increased access to mental health resources via mobile applications and online counselling platforms, enabling services to reach adolescents in familiar digital environments. Additional initiatives included stigma reduction campaigns, resilience-building workshops, and peer-support programs integrated within educational settings and local communities [21]. Collaboration between mental health organizations, community organizations and educational institutions has further embedded these initiatives within schools, universities, and local networks.

Evidence regarding the long-term effectiveness and sustainability of these interventions remains limited, particularly in relation to post-pandemic youth suicide prevention outcomes [39].

##### Interpretive Synthesis

Overall, available evidence suggests increasing psychological vulnerability among young populations in several countries, particularly among adolescent and young females in the post-pandemic period. Although trends vary across countries and available data remain heterogeneous, these patterns highlight the growing recognition of youth mental health within broader national suicide prevention frameworks.

#### 3.2.5. Non-Suicidal Self-Injury

##### Empirical Evidence from Selected Countries

Non-suicidal self-injury (NSSI) represents an important area of concern in youth mental health research [40]. However, data on NSSI remain limited in many countries, since relevant studies during the pandemic and in the post-COVID-19 period have not yet adequately addressed this topic [41].

Research from several European countries, including Spain and Italy, as well as from non-European settings such as South Korea, has reported increased rates of NSSI, especially among younger populations [42]. These results also explore possible associations between NSSI and social and economic stressors, as well as challenges experienced during the pandemic [42]. Studies in the pre-COVID period also showed increased occurrence of NSSI in adolescents with mental disorders, adverse childhood experiences, female gender, low health literacy, exposure to bullying, difficulties in adjustment and physical symptoms [42,43].

Numerous countries have introduced interventions aimed to enhance access to mental health services for young populations experiencing self-injurious behaviours, including suicidality [44]. In England, where approximately one in five females aged 16–24 years reported NSSI, access to mental health services was reported as comparatively limited relative to the reported prevalence of need [26]. In response, national mental health initiatives in the UK have continued investment in mental health services through the NHS Long Term Plan, including actions to improve access to NHS-funded mental health support for individuals up to the age of 25 years [26].

##### Interpretive Synthesis

Overall, the available evidence highlights a growing but still under-documented burden of NSSI among adolescents and young adults. The findings also underscore significant limitations in surveillance, reporting, and comparative epidemiological data across countries. The evidence further suggests that NNSI is associated with a complex interaction of psychological, social and environmental factors, particularly among vulnerable youth populations. At the same time, increasing policy attention to youth mental health and service accessibility has been observed in several national contexts during the post-pandemic period.

#### 3.2.6. Drug Use and Suicidality in Europe

##### Empirical Evidence from Selected Countries

Substance use and substance use disorders have consistently been associated with increased suicide risk, particularly among men [45,46]. Evidence from several countries suggested that alcohol and drug-related mental health burden increased during the COVID-19 pandemic [45,46].

In response, a number of European countries implemented suicide prevention programs and mental health initiatives that incorporated interventions targeting substance use and related psychological distress [47,48]. These initiatives included crisis hotlines, online support services, public awareness campaigns focused on the risks of substance use for mental health deterioration [47,48]. Additional programs aimed to reduce alcohol and drug use by offering region-specific counseling for high-risk populations and by fostering collaboration between health agencies and local organizations. Moreover, many of these programs included suicidality screenings at addiction clinics and community health centers, as well as advanced training for healthcare providers to recognize and address suicide risk in individuals with substance use disorders [48]. These approaches combined early intervention, community-based mental health services, and crisis support across diverse population groups [48].

##### Interpretive Synthesis

Overall, the available evidence indicates a strong and consistent association between substance use and suicide risk across several European contexts, particularly among men. The findings further highlight that substance use, mental health vulnerability, and suicidality frequently coexist within broader psychosocial and public health challenges. The comparative evidence also indicates increased integration of substance use and suicide prevention components within national mental health initiatives during and after the COVID-19 pandemic.

#### 3.2.7. Documentation and Monitoring

##### Empirical Evidence from Selected Countries

Although reliable surveillance systems have been deemed essential for understanding suicide patterns and evaluating prevention strategies, substantial variation has been reported across countries in the quality, accessibility, and completeness of suicide-related data [49].

Periodic fluctuations in suicide rates have been reported internationally, including the selected European countries [49]. Previous research has examined the possible influence of economic conditions, societal changes, and public health interventions on these temporal trends [49]. Studies have also highlighted the importance of incorporating information on psychiatric and physical comorbidities when examining suicide mortality trends, given the correlation between chronic illness and suicide risk.

Werdin and Wyss [32] underlined the lack of robust systems for documenting and monitoring suicide attempts in many European countries. Although the recording of suicide attempts is methodologically more complex than the documentation of suicide deaths, several researchers have emphasized the importance of standardized definitions and concise monitoring procedures for improving consistency and accuracy in reporting and comparative analysis [32].

Werdin and Wyss [32] further identified persistent challenges affecting suicide prevention strategies across many European countries, including stigma, resource constraints, and structural limitations in mental health and psychotherapeutic care systems. Reported areas of focus within national prevention frameworks included: (a) development of suicide prevention programs for males and the elderly, (b) enhanced collaboration across sectors, various professions and stakeholders, and (c) increased involvement of individuals with living experience of mental health challenges in policymaking processes.

The relatively low suicide rates in the RC compared with other European and Eastern Mediterranean countries have also been discussed in relation to potential underreporting [34]. Although police investigation is mandated for all violent or unnatural deaths in the RC, suicides may remain underreported due to social stigma, religious beliefs, and the absence of a structured psychological autopsy system. The predominance of Christian Orthodox religious affiliation in the RC has also been discussed as a potential socio-cultural factor influencing suicide accurate reporting practices [34]. At the same time, anecdotal observations suggest that attitudes toward suicide within the Church in the RC may have become less rigid in recent years.

International evidence has highlighted the role of legislative and procedural reforms in improving suicide documentation practices. In Ireland, reforms to the Coroner’s Act in 2019 were associated with efforts to reduce stigma linked with suicide classification and reporting [50]. Sweden and the Netherlands have also implemented robust psychological autopsy protocols to improve the investigation and classification of unnatural deaths [51].

Nevertheless, concealed suicides, including deaths classified as accidental, undetermined, or ill-defined, continue to represent an important methodological challenge [52]. Such causes include deaths related to drowning, poisoning, and other external causes where suicidal intent remains uncertain [52]. The prevalence of these classifications varies considerably across countries. Data from Finland and the UK indicate that a substantial proportion of suicides may be classified as accidental or undetermined deaths, potentially contributing to the underestimation of suicide mortality rates [21].

The extent of suicide undercounting is relevant both for international comparisons and for the interpretation of temporal patterns within countries, especially when recording practices or socio-cultural attitudes towards suicide change over time. In several countries, age-, gender-, and cause-specific mortality data also remain accessible only through direct request to national monitoring authorities, whereas aggregate mortality statistics are publicly available in many other European settings.

##### Interpretive Synthesis

Overall, the findings indicate that limitations in surveillance systems, reporting practices, and data accessibility continue to represent important challenges for cross-national comparison of suicide mortality in Europe and effective suicide prevention planning in these settings.

The evidence further suggests that socio-cultural factors, legal procedures, classification practices, and variation in monitoring infrastructure may all influence the accuracy and comparability of suicide statistics across countries. Differences in the recording of suicide attempts, accidental deaths, and undetermined deaths also complicate the interpretation of temporal and geographical trends.

Taken together, these findings highlight the central role of surveillance quality and documentation practices within suicide prevention research and public health monitoring frameworks.

#### 3.2.8. Suicide Prevention and the Role of Media

##### Empirical Evidence from Selected Countries

During the COVID-19 pandemic and the subsequent recovery period, media platforms were widely used to disseminate information about mental health resources and support services. Media reporting has also been recognized as an important factor in influencing public perceptions of suicide and mental health [53].

Research conducted during and after the pandemic has examined the role of media environments in mental health communication and suicide prevention [32,53,54,55]. Several studies suggested that media platforms contributed to raising awareness of mental health challenges, disseminating information about available support resources, and reducing stigma associated with help-seeking behaviours [32,54,55]. Public campaigns and media messaging during this period frequently focused on mental health literacy, emotional wellbeing, and access to support services.

The literature has also explored the potential influence of media reporting practices on suicide-related behaviours and public attitudes [53]. In particular, studies emphasized the importance of language, contextual framing, and presentation of suicide-related content in media coverage [53]. Positive media narratives focusing on recovery, coping, and resilience have additionally been examined within suicide prevention communication strategies.

The expansion of digital and social media platforms has further increased the role of online environments in mental health communication. Social media platforms have been used for peer support, dissemination of mental health information, and discussion of self-harm and suicidality. For example, the Australian #chatsafe initiative was developed to support safer online communication among young people regarding self-harm and suicide [56].

Several studies have also explored the use of social media data for monitoring suicide-related expressions and identifying emerging suicide risks [57]. Online platforms may provide real-time information regarding emotional distress, self-harm discussions, and geographical clustering of suicide-related content, particularly in settings where conventional suicide surveillance systems remain limited [57].

##### Interpretive Synthesis

Overall, the evidence suggests that media environments may influence public awareness, help-seeking behaviour, and communication regarding suicide and mental health. The findings further indicate that digital and social media platforms present both opportunities and challenges within suicide prevention efforts. Potential benefits include increased dissemination of mental health information, peer support, and early identification of suicide-related risk signals. At the same time, concerns related to privacy, digital safety, content moderation, and exposure to harmful online material remain important areas requiring further investigation.

Taken together, the comparative evidence highlights the increasingly prominent role of digital communication environments within contemporary suicide prevention and mental health discourse.

## 4. Limitations

This review focused on a purposively selected group of European countries, and therefore does not provide a comprehensive assessment of suicide prevention policies across all EU member states. Major EU countries such as France, Germany, Italy, Spain, Poland, and the Netherlands were not examined in equivalent depth, reflecting both the focused narrative design of the review and differences in policy comparability, data availability, and surveillance accessibility across jurisdictions. Consequently, the findings should be interpreted as a contextualized comparative synthesis of selected European experiences rather than a complete mapping of suicide prevention strategies across Europe. Future research could expand the comparative framework to include a broader range of European health systems and policy contexts.

Importantly, the review intentionally adopted an RC-centered comparative perspective. This analytical emphasis reflected the RC’s evolving policy landscape, limited surveillance infrastructure, and relative underrepresentation within comparative European suicide prevention literature. Accordingly, the asymmetry in analytical depth across countries was an intentional feature of the review design rather than an unintended imbalance. Comparisons with other European countries were therefore used primarily to contextualize the Cypriot case within broader European suicide prevention developments and policy approaches. Nevertheless, because the review was selective and interpretive in nature, some relevant national policies, epidemiological trends, and prevention initiatives may not have been fully represented.

## 5. Implications

The comparative findings suggested that effective suicide prevention requires long-term, coordinated, and multifaceted approaches implemented within comprehensive national mental health policy frameworks [9,58]. Evidence from countries with established national suicide prevention strategies indicated that sustained and integrated interventions, rather than isolated measures, are associated with stronger continuity in prevention efforts and service coordination. The reviewed evidence also highlighted the importance of cross-sectoral collaboration involving healthcare systems, social services, educational institutions, labour sectors, and community organizations in addressing the multidimensional determinants of suicidality.

Furthermore, illustrative examples of relevant initiatives identified across the selected countries included the expansion of telehealth and digital mental health services, increased availability of substance use treatment facilities, development of crisis helplines, implementation of school-based mental health strategies, and targeted support measures for vulnerable populations, including financial assistance initiatives [38,58].

The findings additionally suggested that socio-economic instability, unemployment, and financial insecurity remain important contextual factors associated with suicide vulnerability, particularly among middle-aged men during periods of societal and economic disruption [22]. In this context, the reviewed evidence highlighted the close interaction between mental health outcomes and broader social and economic policy environments, including dimensions related to employment protection and social support. The findings also indicated increasing recognition across several European countries of the importance of gender-sensitive and age-specific approaches within suicide prevention planning.

Evidence reviewed in relation to youth mental health further suggested increasing psychological vulnerability among adolescents and young adults in the post-pandemic period, particularly among young females. School-based mental health initiatives, community-based support services, digital mental health interventions, and peer-support approaches were increasingly incorporated into national mental health responses in several countries [21,39]. However, comparative evidence regarding the long-term effectiveness and sustainability of these interventions remains limited.

The review additionally highlighted evolving patterns in suicide methods across genders. The findings suggested that access to means, socio-cultural context, and gender-related behavioural patterns may influence method selection. While several European countries have implemented means-restriction policies primarily targeting suicide patterns among men, emerging evidence also pointed to increasing use of violent suicide methods among women in some settings [32,34]. These changing patterns indicate the need for continued research examining gender-specific and socio-cultural dimensions of suicidal behaviour.

Importantly, substantial heterogeneity remains across European countries in relation to surveillance capacity, documentation practices, and accessibility of suicide-related data. Limitations in suicide surveillance systems, underreporting, and variation in the classification of suicide deaths and suicide attempts continue to represent significant barriers to accurate cross-national comparison and evidence-informed policy planning. Concealed suicides, including deaths classified as accidental or undetermined, further complicate interpretation of suicide mortality trends across countries [21,52]. In several national contexts, age-, gender-, and cause-specific mortality statistics also remain accessible only through direct request to national monitoring authorities. Taken together, these findings underscored the importance of strengthening surveillance systems, improving policy coordination, and supporting evidence-informed planning across European mental health systems.

The review also highlighted the increasingly prominent role of media and digital communication environments in suicide prevention and mental health awareness during and after the COVID-19 pandemic. Evidence from several countries suggested that media platforms contributed to dissemination of mental health information, promotion of help-seeking behaviours, and public discussion surrounding mental health difficulties [32,53,54,55]. At the same time, digital and social media environments introduce additional challenges related to privacy, information safety, harmful content exposure, and content moderation. Emerging evidence further suggested that social media platforms may function as supplementary sources for monitoring suicide-related expressions and identifying potential suicide risk patterns in real time, particularly in settings with limited surveillance infrastructure [57].

The RC, alongside several other European countries including Greece, continues to face important structural challenges in relation to suicide prevention policy development, surveillance infrastructure, and systematic monitoring of suicidal behaviours, as comprehensive national suicide prevention strategies remain under development. In this context, comparative insights from countries with more established national strategies may provide useful policy perspectives. Nevertheless, suicide prevention approaches need to remain responsive to national socio-cultural conditions, healthcare system structures, and local epidemiological patterns.

Finally, the findings of this review indicated that strengthening epidemiological monitoring systems, improving coordination across sectors, expanding accessibility to mental health support services, and enhancing the quality and comparability of suicide surveillance data remain important components of suicide prevention efforts across Europe in the post-pandemic period.

## 6. Conclusions

The present focused comparative narrative review examined suicide prevention developments across selected European contexts during and following the COVID-19 pandemic, using the RC as a focal analytical case for comparative reflection due to its evolving policy landscape, limited surveillance infrastructure, and relative underrepresentation within comparative suicide prevention literature. Across the reviewed countries, the pandemic and post-pandemic period were associated with increased policy attention toward population mental health, suicide prevention, and the strengthening of mental health support systems [3,4,5]. In several settings, governments expanded existing mental health initiatives and suicide prevention programs or introduced new measures as part of broader efforts to strengthen mental health services and improve access to support [59].

At the same time, empirical evidence regarding the impact of the COVID-19 pandemic on suicide mortality remained heterogeneous across countries and time periods. While increased psychological distress, anxiety and depressive symptoms, and broader psychological vulnerability were widely reported during and after the pandemic, these trends did not consistently correspond to measurable increases in suicide mortality rates [3,4,5]. Evidence regarding suicidal ideation, self-harm behaviours, suicide attempts, and suicide mortality similarly remained mixed and inconsistent across national contexts [3,4,5]. These indicators should therefore not be interpreted as directly interchangeable measures of population suicide risk.

Overall, the following key messages may be supported by the present review:

- Suicide prevention policy development has received increased attention in several European countries during the pandemic and in the post-COVID-19 period, although implementation and policy maturity remain heterogeneous.
- Robust surveillance systems and high-quality epidemiological data are essential for improving suicide prevention planning, evaluation, and cross-national comparability.
- Comparative experiences drawn from selected European countries suggest that coordinated, long-term, and cross-sectoral interventions may strengthen suicide prevention efforts, particularly in countries where comprehensive national strategies and surveillance infrastructures are still evolving.

## Data Availability

No new data were created or analyzed in this study.

## Supplementary Materials

The following supporting information can be downloaded at: https://www.mdpi.com/article/10.3390/healthcare14111528/s1, Table S1: Principal evidence sources included in the narrative review and their contribution to the comparative synthesis intentionally. The data are selective and illustrative, consistent with the focused comparative narrative design of the review. It summarizes the principal categories of evidence informing the thematic synthesis rather than providing an exhaustive inventory of all included sources.
