# Supplementary material for: Suicide Prevention as a Pillar of Sustainable Mental Health: A Focused Comparative Narrative Review of the Republic of Cyprus and Selected European Countries in the Post-COVID-19 Era
Source: Healthcare (Basel). 2026 Jun 1;14(11):1528. doi: 10.3390/healthcare14111528 (PMC13257276; doi:10.3390/healthcare14111528)
Supplement: Supplementary file 1 [file healthcare-14-01528-s001.zip › healthcare-4301608-supplementary.pdf]

Table 1. Principal evidence sources included in the narrative review and their contribution to the comparative synthesis intentionally. The data are selective and illustrative, consistent with the focused comparative narrative design of the review. It summarizes the principal categories of evidence informing the thematic synthesis rather than providing an exhaustive inventory of all included sources.

Source [citation]	Country / Setting	Context	Source Type	Primary Contribution to the Review
International reports				
WHO and European Commission mental health and suicide prevention reports [15]	European Union / International	National suicide prevention strategies	International policy and surveillance (technical) guidance reports	• Synthesised global national strategies; identified core components, indicators, and implementation examples for suicide prevention frameworks.<br>• Supported comparative discussion of European suicide prevention priorities, surveillance systems, and post-pandemic mental health responses.
European Commission [28]	Germany (EU)	COVID-19 state aid measures	Policy database/report	• Documented economic support interventions for individuals and businesses during COVID-19.<br>• Supported the comparative discussion on financial supportive measures and mental health resilience.
European Monitoring Centre for Drugs and Drug Addiction (EMCDDA) [48]	European Union / International	Substance use and COVID-19	International surveillance & trend reports	• Documented the impact of the pandemic in relation to substance use on healthcare services and mental health.<br>• Supported the comparative synthesis on the link among substance use and suicidality.
WHO [53]	International	Media reporting on suicide	Guidance document	• Provided best practice guidelines for safe media communication about suicide.<br>• Supported the comparative discussion on the role of media in suicide prevention.
Government policy document				

UK Department of Health and Social Care [16]	England/ United Kingdom	5-year cross-sector suicide prevention strategy in England	National policy strategy	• Outlined a multi-sector national prevention plan; emphasises data systems, community interventions, and system coordination.<br>• Provided evidence on cross-sector suicide prevention planning, priority groups, crisis support systems, and surveillance-oriented interventions in the post-COVID period.
Swedish National Strategy for Mental Health and Suicide Prevention [17]	Sweden	National mental health and suicide prevention strategy	National policy framework	• Described integrated youth mental health approach and national prevention structures.<br>• Contributed evidence regarding integrated monitoring systems, seasonal prevention approaches, and indicator-based implementation mechanisms.
Finnish Institute for Health and Welfare. [18]	Finland	National suicide prevention programme *2020–2030*	National prevention program	• Provided detailed coordinated prevention measures, surveillance systems, and intervention development.<br>• Provided comparative evidence on structured national suicide prevention planning, measurable objectives, and gender-sensitive interventions.
Nordic overview of suicide prevention efforts [19]	Nordic countries	Regional suicide prevention overview	Comparative regional report on prevention strategies	• Compared Nordic approaches and highlights best practices and cross-country collaboration.<br>• Supported the comparative analysis of suicide prevention structures and monitoring practices across Nordic countries.
UK Office of National Statistics [21]	United Kingdom	England & Wales national suicide statistics	Government statistical bulletin/ Surveillance report	• Provided official suicide registration trends and demographic breakdowns.<br>• Supported the comparative analysis on age- and sex-related suicide trends.
Public Health England [39]	United Kingdom	School mental health approaches	Guidance report	• Promoted whole-school strategies for mental health and early intervention

Epidemiological studies/ Empirical studies				
Chatzittofis et al. [20]	Republic of Cyprus	Epidemiological study	Suicide mortality trends 2004-2020	• Provided primary epidemiological evidence on suicide mortality trends, gender differences, suicide methods, and seasonal variation within the Republic of Cyprus.
Reeves et al. [22]	Europe & North America	Economic recession and suicide	Ecological econometric study	• Provided evidence on the link between economic crisis, unemployment and suicide rates.
Basta et al. [24]	Greece (Crete)	Economic crisis impact on suicide	Observational epidemiological study	• Contributed evidence regarding associations between economic crises, unemployment, gender, and suicide mortality trends & adverse mental health outcomes.
Negash et al. [25]	Germany	University students during COVID-19	Cross-sectional survey	• Provided evidence on the link between financial stress and anxiety and depressive symptoms.
Rhead et al. [26]	United Kingdom & multi-country	Long COVID and finances	Longitudinal population study	• Demonstrated financial deterioration linkage to long COVID and associated mental health burden.
Pope & Hourston [27]	United Kingdom	COVID-19 economic support	Policy analysis/report	• Reviewed government financial support schemes during pandemic and population coverage<br>• Provided documentation on financial support measures.
Su et al. [29]	International	Domestic violence & digital interventions	Public health/tech review	• Examined technology-based mental health interventions for DV survivors during COVID-19.
Morovatdar et al. [30]	Eastern Mediterranean	Suicide methods	Systematic review/meta-analysis	• Identified most common suicide methods across EMR and regional variation patterns.
Bachmann [31]	International	Suicide epidemiology	Narrative review	• Summarised epidemiological patterns and psychiatric risk factors for suicide.
Werdin & Wyss [32]	Germany/Austria/Switzerland	Comparative public health literature/ suicide	Qualitative study	• Explored system-level barriers and facilitators in DACH region prevention efforts.

		prevention systems		• Supported analysis of suicide surveillance systems, underreporting, structural barriers, and prevention policy implementation across Europe.
Hawton et al. [33]	United Kingdom & Ireland	Paracetamol pack size regulation	Comparative policy impact study	• Provided data on the link between overdose deaths and restriction of medication pack size.
Eurostat [34]	European Union	Suicide mortality trends	Statistical release	• Documented suicide rates across Europe over a decade.
Fossi Djembi et al. [35]	France	Suicide prevention programme (VigilanS)	Programme evaluation study	• Provided data on the association between repeat suicide attempts and brief contact interventions.
Wang et al. [36]	China (special population)	Sunlight exposure & mental health	Observational study	• Linked reduced sunlight exposure to adverse mental health outcomes and suicidality.
Bertuccio et al. [37]	Europe	Suicide trends 1990–2022	Comparative epidemiological analysis	• Analysed sex/age patterns and long-term suicide mortality trends.
Warnick & Kolade [38]	USA	Adolescents post-COVID	Narrative review	• Highlighted youth mental health burden and suicidal ideation in the post-pandemic period.
De Luca et al. [40]	Europe/International	NSSI in adolescence	Systematic review/meta-analysis	• Examined developmental patterns and risk factors for non-suicidal self-injury.
Deng et al. [41]	International	NSSI during COVID-19	Meta-analysis	• Estimated prevalence and identified COVID-era risk factors for non-suicidal self-injury.
Cheng et al. [42]	International	Self-harm during pandemic	Systematic review/meta-analysis	• Provided data on the global prevalence in self-harm during the COVID-19 period.
Wang YJ et al. [43]	International	NSSI risk factors	Meta-analysis	• Identified psychological and demographic risk factors for adolescent non-suicidal self-injury.
McManus et al. [44]	England	Self-harm prevalence	Repeated cross-sectional surveys	• Documented trends in self-harm and service contact over time .

				• Provided evidence regarding non-suicidal self-injury trends among young populations and associated psychosocial risk factors.
Czeisler et al. [45]	USA	Mental health during COVID-19	CDC surveillance study	• Provided evidence on the association between suicidal ideation and mental health distress during the pandemic.
Ornell et al. [46]	Brazil/global	Substance use during COVID-19	Narrative/psychiatric review	• Provided evidence on the association between substance use and mental health during the pandemic.
Dawson et al. [47]	Europe	Drug use epidemiology	Public health review	• Reviewed patterns of drug use and implications for interventions.
Lovero et al. [49]	International	Suicide in global mental health	Review article	• Discussed suicide burden and integration into global mental health systems.
Rochford et al. [50]	Ireland	National suicide prevention	Policy/practice paper	• Described Ireland's public health approach to suicide prevention.
Snowdon & Choi [52]	International	Suicide undercounting	Conceptual/review article	• Highlighted systematic underestimation and data gaps in suicide reporting.
Bello et al. [54]	Europe/Spain	Media discourse & COVID-19 suicide	NLP content analysis	• Examined media framing and association with suicide narratives
Marzano et al. [55]	United Kingdom	COVID-19 media coverage	Content analysis	• Analysed media speculation and framing of suicide during pandemic.
Orygen (#chatsafe) [56]	Australia/International	Online suicide communication	Guidance framework	• Provided digital safety guidelines for youth suicide-related communication.
57. Wang et al. [57]	Japan	Social media surveillance	Time-series ML study	• Provided evidence on the link between deep learning and monitor of suicide-related social media trends.
